# Sustainable weight of ear-borne devices for cattle

**DOI:** 10.1093/tas/txaf055

**Published:** 2025-05-03

**Authors:** Emma N Macon, Hope A de Avila, Karen L Launchbaugh, Gordon K Murdoch

**Affiliations:** University of Idaho, Moscow, 83843, USA; University of Idaho, Moscow, 83843, USA; University of Idaho, Moscow, 83843, USA; Washington State University, Pullman, 99164, USA

**Keywords:** Cattle, ear-borne devices, ear health, ear tag, tag weight

## Abstract

Approaches to precision agriculture are increasingly being applied to enhance animal husbandry through data collection and trend analysis. A growing number of technologies involve ear-borne devices such as EID’s (i.e., Electronic Identification tags), health indicators, and location trackers. However, limited research has been conducted to determine how much weight cows’ ears can sustain for a prolonged period of time. Our objectives were to determine if tag weight affects the ear healing rate, mobility, or orientation. Three tag weights (62, 89, and 124 grams) were compared to an unweighted tag (14 grams), using a commercial, two-post ear-tag (i.e., an EnduroTag). These weights were observed daily on 17 dairy and 17 beef cows for six weeks to assess overall ear health (i.e., tissue inflammation), continued mobility of the ear (i.e., movement and twitching), and severity of ear droop. Overall, the results indicate there is a significant difference (*P < 0.05*) in acute irritation, droop, and mobility between the four weight treatments examined. Furthermore, the final observation evaluating the degree of healing after six weeks also showed a significant difference *(P < 0.05)* between tag weights. This information regarding the healing outcome, orientation, and mobility of the ear is valuable to the growing suite of ear-borne technologies.

## Teaser

Ear-borne technologies for livestock are a growing market and information regarding the healing process, mobility, and orientation of the ear associated with added weight to the ear is extremely valuable. Added weight impedes the healing, mobility, and orientation of the ear, but minimal weight paired with a period of healing can be sustainable.

## INTRODUCTION

### Ear-Borne Technology

Electronic technologies are increasingly being used in livestock management to improve animal husbandry. Many of these technologies are being placed on animal ears to facilitate animal identification, health monitoring, and location tracking. The current forms of visual animal identification and disease vaccination verification (e.g., Bangs tags) are being replaced by electronic Radio Frequency Identification (RFID) tags (Schleppe et al., 2010; APHIS, 2014). Disease traceability and data recall of individual animals and herd information are also facilitated by RFIDs. Health monitoring devices placed on the ear are increasingly being used in dairies and feedlots to report body temperature and movement data (Lee and Seo, 2021). If an animal defers from its baseline, a manager can be notified, and examination or treatment can be accomplished. Location trackers on the ear are also becoming available. These devices collect data that can be used to track land utilization or allow managers to create boundaries within the device’s platform which will then trigger an alert if an animal leaves that boundary (Trotter, 2019). Despite their varying purposes and capabilities, the commonality of these ear-borne technologies is their added weight.

### Existing Research

In the late 1990s and early 2000s, some of the first studies to test the effectiveness of an ear-based electronic invisible fence, did so with a single punch tag weighing 113 grams (Tiedemann et al., 1999). Before weighted tags were applied, a hole was punched in the animal’s ear and allowed to heal. These tags were left in the ear for 13 to 14 d and irritation such as swelling was observed once the tags were removed but further details were not disclosed. Tiedemann and colleagues (1990) speculated that ear-borne devices need to weigh no more than 25 grams.

In a study conducted to design a Global Navigation Satellite System (GNSS) to be used in a feedlot setting, an ear tag device was developed (Schleppe et al., 2010). Several devices with weights ranging from 114 to 250 grams were tested. In a preliminary study, they determined that the cow ear could withstand up to 250 grams for an undisclosed short amount of time. However, in a later study, it was found that a device weight of 227 grams was intolerable for more than 3 to 5 d by most cattle tested, but 114 grams was tolerated for up to 20 d (Schleppe et al., 2010). Although challenges regarding the tolerance of the tag weight were mentioned, this study made little reference as to what tolerability meant in terms of ear health.

Technological advances have led to lighter weight devices. A more recent study compared a 32-gram device to a standard tag (8 grams) for 11 mo on three groups of animals all under 30-mo of age (Gobbo Oliveira Erünlü et al., 2023). The condition of animals’ ears was observed for redness, swelling, pain reactions, and crusting (i.e., blood or pus discharge around tag posts). All animals experienced notable redness, occasionally exhibited pain responses and crusting observed on both types of tags, and no swelling was observed (Gobbo Oliveira Erünlü et al., 2023). This study provided ear health data for a device weighing 32 grams aligning with many of the commercial products that generally weigh up to 35 grams.

### Objectives

As ear-borne devices become more common in livestock management, the effect of added weight on the ear needs to be examined and considered. Research comparing a range of weights or the potential value of healing periods has been limited. Our research objectives were to: 1) determine how 2–post bovine ear tags of varying weights affect the ear healing outcome, mobility, and orientation; and, 2) determine if a 2-wk healing period before applying weighted ear tags would affect levels of irritation. We anticipate as weight increases there will be more extensive irritation, mobility restrictions, and abnormal ear orientations while the control group will exhibit a minimal amount of these criterion. Furthermore, we expect a 2-wk healing period will be effective in improving the extent of healing before additional weight is applied.

## MATERIALS AND METHODS

We conducted two experiments to examine responses across a range of weights on an ear. First, we examined if adding weight to standard ear tags affected ear health and condition. In our second experiment, we considered if implementing a healing period before adding weight to the ear tag affected ear health.

### Experiment 1

**Figure 1.**
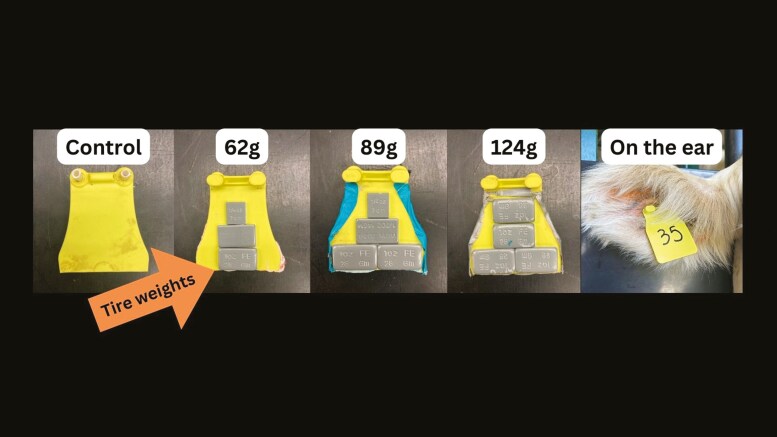
Cattle ear tags representing the four treatments constructed by adding tire weights to 2-pronged ear tags to examine ear response to weighted tags. Tags were deployed between the two cartilaginous ridges about 5.2 cm from the point of attachment to the head.

The research animals in this experiment included 17 mature beef cows, breeds including Hereford (averaging 4.4 yr old and 1405 lbs), Angus (averaging 4.3 yr old and 1390 lbs), and Wagyu (averaging 4.5 yr old and 1215 lbs) and 17 mature Holsteins (averaging 2.2 yr old and 1255 lbs). This research followed the Animal Care and Use Committee protocols of the University of Idaho (IACUC-2023-31). Research was conducted at the Washington State University Cattle Feeding Laboratory in Pullman, WA. Animals were assigned to four treatment groups (7-9 cows in each) with varying ear tag weights. Increased tag weight was accomplished with steel adhesive tire weights (CKAuto, Zhejianag, China) applied to 2-pronged ear tags (EnduroTag, Victoria, Australia; Figure 1). Tags were deployed between the two cartilaginous ridges about 5.2 cm from the point of attachment to the head. The four treatments consisted of a control group of 14 grams (n = 7; tag without additional weight), 62 grams (n = 9), 89 grams (n = 9), and 124 grams (n = 9) which were worn during the 6-wk trial.

Throughout this trial, animals’ ears were regularly observed for irritation, mobility restrictions, and abnormal ear orientation by a singular person as to ensure observations were consistent. The data collected was qualitative. The identifiers of irritation included the presence of dried or fresh blood or, puss, and swelling (assessed by ear thickness around punch holes and tactile temperature). Irritation observations were conducted weekly when animals were restrained in a chute to allow for close inspection. Irritation was observed over a 5-wk period with the final healing assessment taking place in the sixth week. Mobility was assessed three times per week over a period of six weeks by watching how fully the animal could twitch and flex the treatment ear in comparison to their non-treatment ear. The predetermined categories of mobility observations included full mobility, slightly reduced, significantly reduced, and no mobility. Orientation was judged by the extent the treated ear’s relaxed position differed (droop) from the animal’s other ear, three times per week over the course of six weeks while animals were in a feeding pen with other animals. The categories of orientation included no droop, slight, moderate, and significant droop.

The degree of healing was assessed and scored on the final day of the experiment. During this observation, all tags were removed from the ear, and punch holes were cleaned. Crusting, fresh blood, and scar tissue were each scored on a scale of 1 through 3 with increasing severity (Table 1). Crust was described by Gobbo (2023) as dried exudate composed of serum, blood, and or puss and scoring occurred before cleaning. After cleaning, fresh blood and scar tissue were scored. Scar tissue was scored based on whether the ear was trying to heal over the backing (plastic bar 4.1 cm long, 1.3 cm wide) of the ear tag.

**Table 1.** A healing assessment after cattle were fitted with weighted ear tags (Experiment 1: 6 wk and Experiment 2: 2 wk) was conducted. The criteria observed were scored on a scale of 1 through 3 with increasing severity. Each of the two tag posts received an individual score for each criterion combined for an overall score. The lowest score possible was 6 and the highest score possible was 18. The higher scores correlated with a lower degree of healing.

*Criteria*	*Severity*	*Score*
Crust	None	1
	Crust	2
	Severe crust	3
Scar tissue	Not covered	1
build up over	Partially covered	2
tag backing	Fully covered	3
Fresh blood	None	1
	Some	2
	Severe	3

Irritation, mobility restrictions, and abnormal ear orientation were summarized as the number of times each occurred within each weight treatment each week, for six weeks. Because the data collected were qualitative, we used non-parametric analysis methods to derive our results. A Chi-square test was applied to each observation category for each week of the experiment and tested against an alpha level of 0.05 (Privitera, 2012). Additional Chi-square pairwise analyses were conducted for irritation between each of the weight treatments and corrected for multiple comparisons with Bonferroni’s Correction for a new alpha level of 0.0083 (0.05/6; Privitera, 2012). For the final healing assessment, a Kruskal-Wallis H test (Kruskal and Wallis, 1952; Privitera, 2012) was applied to rank healing scores within each treatment to examine the distribution of healing. Dunn’s test was used as a post hoc analysis (Dinno, 2015) for pairwise comparison for each of the treatment groups with Z values converted to *P* values. Bonferroni’s Correction was used again to adjust the p-value.

### Experiment 2

Research at the University of Idaho (UI) Beef Center in Moscow, ID following the Animal Care and Use Committee protocols of the University of Idaho (IACUC-2023-31). The research herd included 16 mature Charolais cows (averaging 4.75 yr old) weighing on average 1557 pounds. These animals were assigned to one of two treatments: 1) standard 2-prong Endurotags weighing 14 grams (tag without additional weight) deployed two weeks before a total weight of 63 grams was applied; and 2) a total weight of 63 grams was applied without a healing period. All animals had weighted tags attached for two weeks. Placement and weighted tag design was the same as experiment 1.

A healing assessment (as in experiment 1) was conducted two times over the course of this four-week experiment. The first assessment of group 1 after the 2-wk healing period when standard tags were removed and before the weighted tags were applied. The second assessment occurred on the final day of the experiment and all animals received a healing score. The scores assigned in the field were appointed by a singular person for each observation. Additionally, 4 photos were taken of each animal (front and back of ear, before and after tag was removed). A panel of three personnel observed the images of animal ears that had tied scores with no knowledge of what treatment group the animals were in and collaboratively scored ears from most to least healed. This panel followed the criteria of crust, scar tissue, and fresh blood in Table 1 to rank.

A Kruskal-Wallis H test (Kruskal and Wallis, 1952; Privitera, 2012) was applied to rank the healing score within each treatment to examine the distribution of healing. The overall rank of animals was put through a Mann-Whitney U test to validate our ordinal data (Privitera, 2012).

## RESULTS

### Experiment 1

#### Acute irritation.

**Figure 2.**
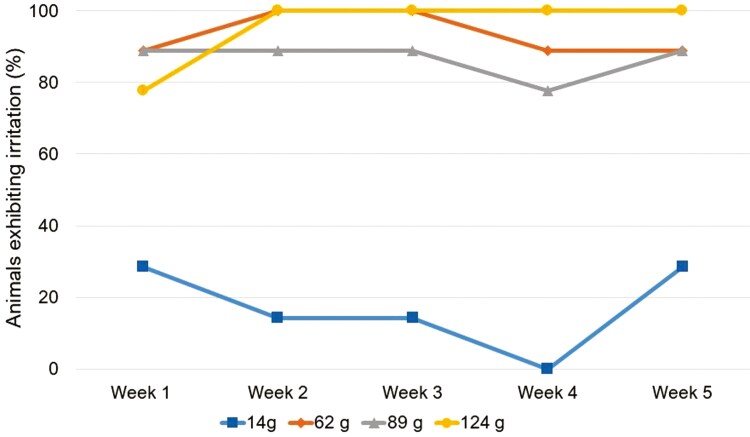
When cattle were fitted with weighted ear tags, all of the added-weight treatments (62, 89, and 124g) exhibited more irritation of the ear than the control treatment (14g). Animals were closely observed in a chute once per week for 5 wk for signs of acute irritation (i.e., blood, puss, and swelling).

Irritation was observed and all weight treatments differed from each other (*P < 0.05*; Figure 2) when compared across the entire 5-wk period. Pairwise comparisons revealed that all weight treatments exhibited more irritation than the control group (*P < 0.008*) but were similar to each other (*P > 0.008*).

#### Droop.

**Figure 3.**
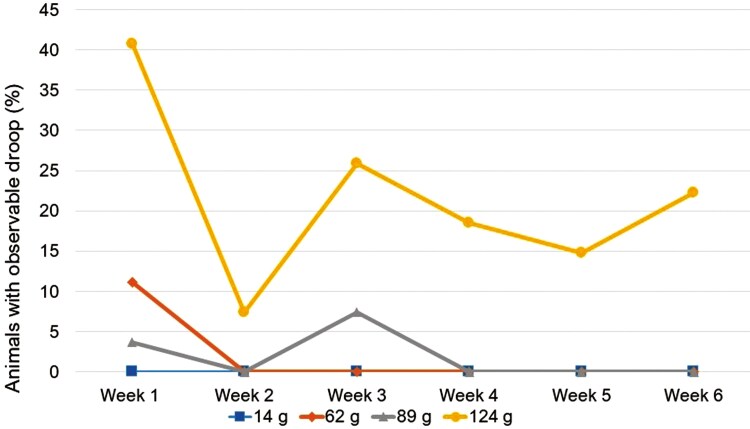
Cattle fitted with weighted ear tags, of varying weight (14, 62, 89, and 124g) all exhibited observable droop as some point during the 6-wk trial. The heaviest weight treatment was the only treatment where cattle consistently exhibited droop. Animals were observed in their pen 3 times per week for 6-wk for abnormal ear orientation at various degrees of none, slight, moderate, and significant.

Overall, weight treatments differed in observable droop (*P < 0.05*). Paired comparisons revealed in the lightest weight (62g), the mid-weight (89g), and control groups on a 6-wk average only 2% or fewer field observations denoted observable droop in each treatment (*P > 0.008*; Figure 3). However, the heaviest weight (124g) had more observable droop with a 6-wk average of 22% of animal observations showing droop (*P < 0.008*).

#### Mobility.

**Figure 4.**
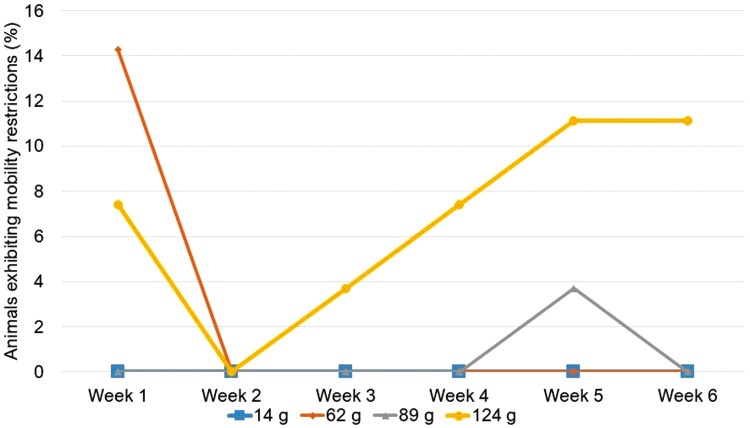
Cattle fitted with weighted ear tags, of varying weight (14, 62, 89, and 124g). Animals in the control group (14g) showed no restricted mobility while other treatment groups had at least one occurrence of restricted mobility; however, the lightest (62g) and mid-weight (89g) groups did not experience consistent signs of restricted mobility. Animals were observed 3 times per week for 6-wk for full mobility, slightly reduced, significantly reduced, and no mobility.

When all treatments were compared, mobility restrictions differed overall (*P < 0.05*). Paired comparisons revealed the lightest (62g) and mid-weight (89g) groups had the least amount of mobility restrictions documented in the field with 6-wk averages of 2% and 1% of animal observations showing restricted mobility and were comparable to the control group (*P > 0.008*) which showed no restricted mobility. The heaviest group exhibited the most mobility restrictions with a 6-wk average of 7% of animal observations showing restricted mobility and differed from all other groups (*P < 0.008*). The lightest group had the second most mobility restrictions due to the high occurrence of mobility issues one week post tag application. However, this occurrence rate dropped to 0 for the remainder of the study (Figure 4).

#### Final healing.

**Figure 5.**
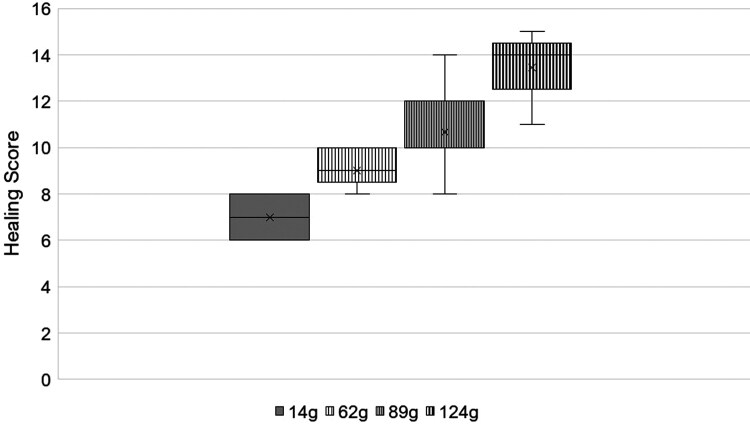
A healing assessment 6 wk after cattle were fitted with weighted ear tags revealed that as weight increased, the degree of healing decreased. Animals were scored on crusting severity, scar tissue buildup, then tags were cut out, and the tag sites were cleaned then scored on the presence of fresh blood. The higher scores correlate with a lower degree of healing. The shaded boxes represent the upper and lower quartiles, the × represents the average, the horizontal bar represents the median, dots represent outliers, and whiskers represent the extremes.

Final healing was assessed after 6 wk by scoring crusting, fresh blood, and scar tissue on a scale of 1 through 3 with increasing severity. Scoring was conducted after tags were removed from the ear. The lowest score an animal could receive was 6, representing greater healing, and the highest was 18, representing little to no healing. As weight increased, the healing scores assigned to each animal also increased, reflecting lower degrees of healing (Figure 5). The average scores of each treatment increased with treatment weight: Control = 7.0 score, 62 g. = 9.0 score, 89 g. = 10.7 score, and 124 g. = 13.4 score.

A Kruskal-Wallis test performed by ranking the healing scores within each treatment group showed differences among the treatment groups (*H = 25.028, df = 3, P < 0.05*). With further comparison revealing the two heaviest weights (89 and 124g) differed from the control group (*P < 0.05*) but not from each other (*P > 0.05*). The lightest weight (62g) was similar to the control and mid-weight groups (*P > 0.05*) but differed from the heaviest group (*P < 0.05*).

### Experiment 2

**Figure 6.**
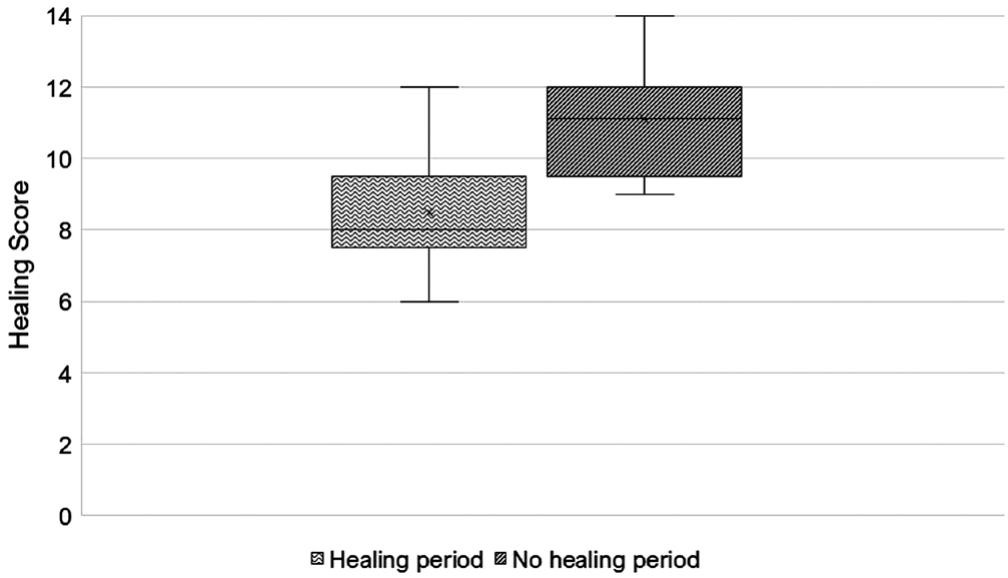
After cattle had weighted ear tags for 2-wk, a healing assessment revealed that a 2-wk healing period before weight application improved healing outcomes. The higher scores correlate with a lower degree of healing. Animals were scored on crusting severity, scar tissue buildup, then tags were cut out, and the tag sites were cleaned then scored on the presence of fresh blood. The shaded boxes represent the upper and lower quartiles, the × represents the average, the horizontal bar represents the median, and whiskers represent the extremes.

This experiment followed the same metrics as experiment 1 but scoring occurred after 2 wk of weighted tag application. Animals that received a 2-wk healing period before weight application had scores that showed less irritation and greater healing than the animals without a healing period before weight was applied (*H = 5.835, P < 0.05*). Ranks assigned to photographs further supported that the group with a healing period before weight application better ear condition than the group without a healing period (*Z=±2.731, P < 0.05*). Of the animals in the pre-healed treatment, 75% showed better condition than the animals without an initial healing window (Figure 6).

## DISCUSSION

As new wearable technologies are developed for cattle, it is important to consider animal irritation and health response to those devices. Ear tags have long been used in the livestock industry and associating devices with this management system are generally well received by producers. This research was intentional in its evaluation of adding weight to dual-posted ear tags and the effect it conferred to the healing and ear positioning of cattle. In this study, lighter ear tags (62g or less) cause lower degrees of abnormal ear orientation, mobility restrictions, and healed more fully in cattle. Across a 6-wk average, only 2% of animals fitted with lighter tags exhibited abnormal ear orientation and mobility restrictions. The lightest weighted tag showed the greatest level of healing among the three weighted treatments and were comparable to the unweighted control tag. Increased weight added to the ear resulted in greater irritation, observations of orientation and mobility restrictions although abnormal orientation and mobility occurred less in all treatments when compared to irritation. In a preliminary study, added weight resulted in a pendulum movement and according to Harmon and colleagues’ mechanical disturbance (movement of wound site) can re-irritate the wound, prolonging the time it takes for it to heal (Harmon et al., 2023). The variations of droop and mobility restrictions from week to week in this study could be the result of slight trauma events on feeders and chutes, occurring on a weekly basis as a result of handling. These animals were fed out of feeders and ran through a chute on a weekly basis replicating typical wear and tear of these tags associated with handling.

Furthermore, the implementation of a healing period before application of a weighted device is supported to favorably reduce irritation and promote better healing. The process of wound healing goes through 3 or 4 phases, depending on how different processes are grouped. Phases typically last over the course of three weeks but can last up to six weeks (Winkler, 2021; Harmon et al., 2023a). We allowed two weeks for healing before adding weight to the ear tag which resulted in better healing in three quarters of the animals with a healing period compared to those without a healing window. Furthermore, in an experiment involving sheep and different ear tag types, it was denoted that “lesion severity” was heightened during the first two weeks after application but declined in the following weeks (Edwards et al., 2001). Tiedemann et al. (1999) also allowed the tag sites to heal before application of weighted devices to the ear for two weeks after which some irritation and swelling were still observed. However, this may have occurred because the heavier weight (113g) applied to the animals (Tiedemann et al. 1999). The time required for the tagging site to heal could vary depending on environmental conditions. In these two experiments, the animals resided in two different facilities; experiment 1 animals were kept in a feedlot like system with round bale feeders and were frequently run through a chute for reasons outside of this study, whereas experiment 2 animals were kept out on pasture and only handled through the chute for observations. Animals in experiment 1 had a higher chance of disturbing the healing process due to trauma events caused by feeders and head catch gates. Despite these differences, a healing period does improve the healing process when applying weight to the ear.

Lastly, an important observation during these experiments was the potential value of airflow between the tag and the ear to promote healing. In a preliminary study, the tag design we used allowed for very little airflow, unintentionally, which caused severe irritation and healing restrictions resulting in the termination of the study. In two studies (Harmon et al., 2023; Gobbo Oliveira Erünlü et al., 2023) it was suggested that increased airflow to the tagging site may allow the ear to heal more quickly and completely. One of the key stages in wound healing is the proliferation stage which is characterized by fibroblasts and capillaries moving to the area of damage to create the framework for cellular regrowth and rebuilding. Before this occurs, tissue granulation or scaffolding must take place (Winkler, 2021). Airflow can aid in this scaffolding but being overly dry can hinder the healing process as well. Furthermore, the accumulation of moisture around the wound can extend bacterial and fungal pathogens thereby delaying healing in the absence of a clean dry surface promoted by airflow.
